# Minimally Invasive Video-Assisted Thyroidectomy and Parathyroidectomy with Intraoperative Recurrent Laryngeal Nerve Monitoring

**DOI:** 10.1155/2009/739798

**Published:** 2010-02-08

**Authors:** Emad Kandil, Shafik N. Wassef, Haytham Alabbas, Paul L. Freidlander

**Affiliations:** ^1^Division of Endocrine and Oncological Surgery, Department of Surgery, Tulane University School of Medicine, New Orleans, LA 70112, USA; ^2^Department of Otolaryngology Head & Neck Surgery, Tulane University School of Medicine, New Orleans, LA 70112, USA

## Abstract

*Objective*. Our goal is to study the feasibility of using intraoperative neuromonitoring (IONM) in minimally invasive video-assisted thyroidectomy and parathyroidectomy (MIVAT/P) with emphasis given to the identification of recurrent laryngeal nerve (RLN). *Methods*. Consecutive series of forty-seven patients with seventy-seven recurrent laryngeal nerves at risk undergoing both MIVAT/P and IONM were enrolled in this retrospective, nonrandomized analysis study. All operations were performed by the same surgeon within an academic institution setting. All patients underwent vocal cord evaluation postoperatively. Demographics and intraoperative and postoperative complications following surgery were collected. *Results*. Out of seventy-seven RLNs, there was one permanent unilateral RLN injury (1.29%) in a patient with advanced papillary thyroid cancer, managed by cord injection. There was another transient RLN paresis that resolved spontaneously (1.29%). There were no instances of equipment malfunction or interference. *Conclusions*. To our knowledge, this is the first reported MIVAT/P series from the United States of America with a standardized IONM technique. The technical feasibility of IONM seems acceptable and may serve as a meaningful adjunct to the visual identification of nerves. Neuromonitoring during MIVAT/P is effective in providing identification of laryngeal nerves and enables surgeons to feel more comfortable with MIVAT/P. Comparative series are needed for further evaluation.

## 1. Introduction

The recurrent laryngeal nerve (RLN) injury during surgeries on thyroid and parathyroid remains the most significant commonly found complication of endocrine surgery in the neck, and it can result in significant morbidity including temporary or permanent paralytic dysphonia and dysphagia. Rates of nerve injury published in the literature typically range from 1% to 2% and are significantly higher for re-operation. Transient injury occurs in approximately 5% with 95% of the injuries recovering normal function [1]. It has long been accepted that anatomic identification of both the RLN and the external branch of the superior laryngeal nerve (EBSLN) is the safest way to reduce nerve injury rates to a minimum [2] and certainly injury rates are lowest when surgery is carried out by experienced endocrine surgeons or thyroid surgeons in specialized centers with high caseloads [3]. Nonetheless, identification and preservation of the RLN do not eliminate the possibility of nerve injury. An anatomically intact nerve may still show altered function postoperatively due to multiple factors, such as neural stretch during goiter retraction. 

## 2. Patients and Methods

This is a retrospective, nonrandomized case series analysis study. The study group comprised of forty-seven patients who underwent thyroid or parathyroid surgery at Tulane University Medical Center from October of 2007 to April of 2009. 

The protocol for assessment of the laryngeal nerves included routine preoperative and postoperative laryngoscopy at one to two weeks. Stroboscopy and electromyography (EMG) assessment were not used in this study.

**Figure 1:**
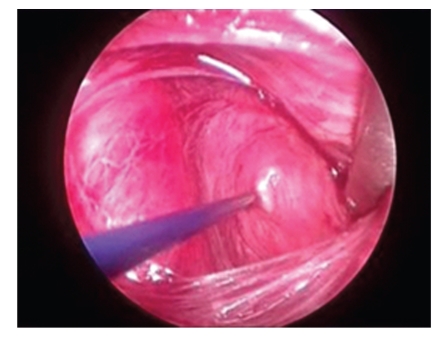
Intra operative nerve monitoring of right recurrent laryngeal nerve.

A standard MIVAT/P gasless approach to the thyroid gland was performed under general anesthesia [4]. The surgeon stands at the lesion side, the camera operator on the other side, and an assistant at the patient's head. We routinely use a 30°5 mm endoscope. All vessel ligation during the procedure was done using the harmonic scalpel (Ethicon, NJ). Additionally, the harmonic scalpel was also used to divide the isthmus in lobectomies, to isolate the gland from the trachea. After routine attempted anatomical observation of the RLN, stimulation of the nerve was done with a hand-held nerve stimulator with either the Medtronic Varistim III Nerve Stimulator (Medtronic Inc., Minneapolis, MI, USA) or the Nervona system (Nervona, CA, USA) at currents of 1 to 2 mA on completion of dissection [2, 5]. The RLN was stimulated at the most proximal exposed site of the nerve (Figure 1).

## 3. Statistical Analysis

Data for continuous variables are expressed as median and range, unless specified otherwise. The outcome measures primarily the morbidity and mortality. Statistical analysis was computed with MS Excel 2007 for Windows. All efforts were made to avoid sources of bias.

## 4. Results

A total of seventy-seven nerves in forty-seven patients were included in this study. The mean age was 50.19 years (range 14–79). There were thirty-seven females (79%). There were twenty-three total thyroidectomies (48.9%), nineteen hemithyroidectomies (*41.3*%), 10.6% and ten parathyroidectomies *(10.6*%*)*.

Out of seventy-seven RLNs, there was one permanent unilateral RLN injury (1.29%) in a patient with advanced papillary thyroid cancer managed by postoperative cord injection. There was another transient RLN paresis that resolved spontaneously (1.29%). 

No bilateral vocal cord paresis or paralysis occurred. There were no instances of equipment malfunction or interference and 0% mortality. 

**Figure 2:**
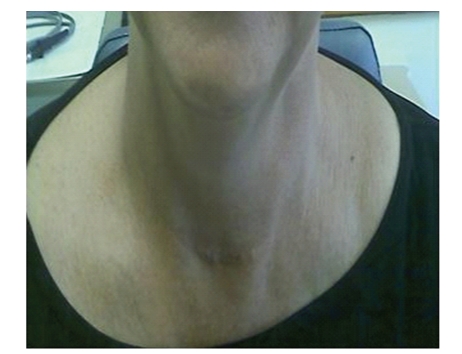
Excellent wound healing one week postoperatively.

There was no other perioperative morbidity. No hemorrhage or cervical hematoma was observed, and none of our cases developed wound infection (Figure 2). 

## 5. Discussion

One of the most common operations throughout the world is thyroidectomy, and it has a low morbidity rate if performed by skilled surgical teams [6]. Conventional thyroidectomy requires a transverse cervical incision that leaves a visible scar on the anterior surface of the neck. The application of minimally invasive techniques for thyroid surgery was primarily motivated by the attempt to improve the cosmetic result of this operation. The aesthetic point of view is particularly important for young women, who constitute a large part of patients affected by thyroid diseases. Nonetheless, minimally invasive surgery should also guarantee better postoperative outcomes. Most MIVAT/P allows for a prompt postoperative recovery and is performed as an outpatient procedure in some clinical settings [7]. It has been recently demonstrated that endoscopic [8] and video-assisted [9–11] procedures for thyroid and parathyroid surgery have some advantages over conventional methods of surgery in terms of not only cosmetic results but also analgesic requirements and postoperative recovery. 

On the other hand, it should be pointed out that these procedures are not easy or feasible in all clinical settings. They are technically demanding and require a surgical team skilled in both endocrine and endoscopic surgery. The endoscopic and video-assisted procedures require learning and a training period that can be time consuming, especially at the beginning of the experience. However, with training, learning, and experience, along with the continuous development in scientific medical technologies, MIVAT/P will be done in more centers and more candidates will be qualified for these kinds of surgeries [12].

The recurrent branch of the laryngeal nerve (RLN) innervates all of the laryngeal muscles except the cricothyroid muscle, which regulates the tension of the vocal cords and is innervated by the external branch of superior laryngeal nerve. If damage to the RLN is *unilateral*, the patient may present with voice changes including hoarseness. *Bilateral* nerve damage can result in breathing difficulties and aphonia, the inability to speak. The right recurrent laryngeal nerve is more susceptible to damage during thyroid surgery due to its relatively medial location. The nerve injury in this series was on the right side.

Variations in the expected anatomic position of the RLN can occur. The risk of nerve injury increases in patients with anomalous RLN anatomy. Such anomalies include nonrecurrence of the RLN, RLN displacement by thyroid nodularity or paratracheal lymphadenopathy, extralaryngeal branching of the RLN, and variations of the nerve course in relation to the inferior thyroid artery and ligament of Berry. In a recent analysis of RLN anatomy in 491 thyroid surgery cases, 60.8% of RLNs were found in the expected tracheoesophageal groove position, whereas 4.9% were lateral and 28.3% were posterior to the trachea. Of greatest concern are those cases where the RLN is found on the anterior surface of the thyroid gland, a particularly high-risk area for nerve injury [13]. The surgical importance of this nerve relates to its proximity to the superior thyroid vessels.

To preserve the RLN, most surgeons tend to avoid rather than expose the nerve, suggesting selective ligation of the upper pedicle vessels. Visualization of the RLN always should be achieved. 

Miccoli and colleagues introduced the minimally invasive video-assisted thyroidectomy procedure in 1998 [14]. During MIVAT /P, most surgeons do not use IONM to search for the RLN. In our series consisting of forty-seven operations, we were able to visualize the RLN in 100% of the cases. Among these, the nerve ran entirely medial and separate to the superior thyroid artery in most of the patients. It crossed the artery or its branches in the remaining patients.

IONM of the RLN during thyroid and parathyroid surgeries has been claimed in some studies to reduce the rate of nerve injury [15, 16]; however, other studies have shown it to be of no benefit and no reduction in the risk of RLN injury [17–20] compared with anatomic identification. However, Hermann et al. showed that IONM could be useful in identifying the RLN especially when anatomically altered by prior surgery or large masses [21]. Numerous studies have shown that only routine exposure of the RLN is associated with very low rates of injury in high-volume centers (less than 0.3%) [22].

During MIVAT/P, the surgical incision is really small in most cases to allow palpation of the nerve or the larynx as methods of identification. In our institution, we have been using IONM as a method of RLN identification during MIVAT/P operations. Now we are reporting our experience retrospectively. At the beginning of implementing both technologies together, we hypothesized that this method of monitoring the RLN would decrease/prevent the risk of iatrogenic injury with lesser risk to the patient. We aimed to combine the potential advantages of IONM to MIVAT/P. Our initial clinical results were very promising showing that the use of IONM is easy and feasible in MIVAT/P, and it was rather safe with no intraoperative complications or conversion to traditional open approach. It gives the surgeon more confidence during MIVAT/P procedures. Though the operating space in these kinds of endoscopic surgeries is relatively narrow, there was no difficulty using the IONM simulator probe. RLN was identified in each surgical step. The tip of the monopolar probe is flexible, which helped the surgeon to go beyond the view of his field. This stimulator was helpful in detecting the plane of dissection. During surgery, the RLN may get injured while it still looks intact without the nerve being actually cut. This can be caused by different sorts of injuries, which include, but are not limited to, traction, pressure, ischemia, ligation, crush, electrical or suction injury [23, 24]. The role of IONM is not only during surgical dissection. Its role extends after the operation as it can help detect how the RLN is functioning at the end of the procedure. This provides the opportunity to stage contralateral surgery in case of suspected RLN injury, thus avoiding the risk of RLN injury bilaterally. We also would like to mention that in our experience, we never had device failure or any unexpected events with the use of IONM during MIVAT/P.

Our experience of IONM with MIVAT/P showed that IONM could be a very useful adjunctive method for RLN identification besides endoscopic visualization and magnification. This possibly will decrease RLN injury and improve the quality and outcomes of MIVAT/P operations [25]. Kern [26] discussed an important point that nerve monitoring during surgeries can possibly decrease the medico-legal liability. However, the literature needs a study with a wider scale of patients to accurately prove our citations. To show a decrease in RLN paralysis rates from 2% to 1% per nerves at risk, a study group of approximately 1,000 patients would be necessary [27]. However, this requires more training and experience. Standardization of the technique and wider availability of these technologies in centers performing these kinds of operations would be of great benefit.
